# Faithful *SGCE* imprinting in iPSC-derived cortical neurons: an endogenous cellular model of myoclonus-dystonia

**DOI:** 10.1038/srep41156

**Published:** 2017-02-03

**Authors:** Karen Grütz, Philip Seibler, Anne Weissbach, Katja Lohmann, Francesca A. Carlisle, Derek J. Blake, Ana Westenberger, Christine Klein, Anne Grünewald

**Affiliations:** 1 Institute of Neurogenetics, University of Lübeck, 23562 Lübeck, Germany; 2 MRC Centre for Neuropsychiatric Genetics and Genomics, Institute of Psychological Medicine and Clinical Neurosciences, School of Medicine, Cardiff University, Cardiff CF24 4HQ, United Kingdom; 3 Molecular and Functional Neurobiology Group, Luxembourg Centre for Systems Biomedicine, University of Luxembourg, L-4367 Belvaux, Luxembourg

## Abstract

In neuropathology research, induced pluripotent stem cell (iPSC)-derived neurons are considered a tool closely resembling the patient brain. Albeit in respect to epigenetics, this concept has been challenged. We generated iPSC-derived cortical neurons from myoclonus-dystonia patients with mutations (W100G and R102X) in the maternally imprinted *ε-sarcoglycan (SGCE*) gene and analysed properties such as imprinting, mRNA and protein expression. Comparison of the promoter during reprogramming and differentiation showed tissue-independent differential methylation. DNA sequencing with methylation-specific primers and cDNA analysis in patient neurons indicated selective expression of the mutated paternal *SGCE* allele. While fibroblasts only expressed the ubiquitous mRNA isoform, brain-specific *SGCE* mRNA and ε-sarcoglycan protein were detected in iPSC-derived control neurons. However, neuronal protein levels were reduced in both mutants. Our phenotypic characterization highlights the suitability of iPSC-derived cortical neurons with *SGCE* mutations for myoclonus-dystonia research and, in more general terms, prompts the use of iPSC-derived cellular models to study epigenetic mechanisms impacting on health and disease.

Induced pluripotent stem cell (iPSC) technology has greatly advanced our understanding of pathways underlying neurological disorders^1^,^2^. Recent work suggested epigenetic variations between different iPSC lines from the same individual and even between different passages of an identical clone^3^, raising doubt as to the applicability and reproducibility of the method. Therefore, it is of great importance to investigate the utility of iPSC technology to study specific epigenetic-related neurological diseases such as myoclonus-dystonia (M-D).

M-D is an alcohol-responsive early-onset movement disorder. Patients commonly suffer from myoclonic jerks of the neck, trunk, and upper limbs, as well as dystonia. Established diagnostic criteria classify patients as ‘possible’, ‘probable’, and ‘definite’ M-D. More than 75% of the clinically ‘definite’ cases harbour mutations in *SGCE*^4^.

*SGCE*-associated M-D is inherited in an autosomal-dominant fashion with variable expressivity and incomplete penetrance^5^. The latter is caused by maternal imprinting, an epigenetic phenomenon resulting in selective silencing of the maternal *SGCE* allele^6^,^7^, a finding that is of translational relevance as it specifically informs genetic counselling of mutation carriers.

The protein encoded by *SGCE*, i.e. ε-sarcoglycan, belongs to a family of transmembrane proteins. A brain-specific isoform of *SGCE*, which includes an additional exon, i.e. exon 11b, is predominantly expressed in the cerebral cortex, the cerebellum, and the hippocampus^8^.

While wildtype ε-sarcoglycan localizes to the plasma membrane, all missense mutant forms currently analysed were retained intracellularly and degraded by the proteasome in overexpression studies using mammalian cell lines^9^.

Here, we explore, for the first time, the potential of iPSC-derived neurons from M-D patients as a human cellular disease model to study the molecular consequences of endogenous *SGCE* mutations, with special emphasis on the phenomenon of maternal imprinting of the *SGCE* gene.

## Results

### iPSCs from M-D patients with *SGCE* mutations are efficiently differentiated into cortical neurons

**Figure 1:**
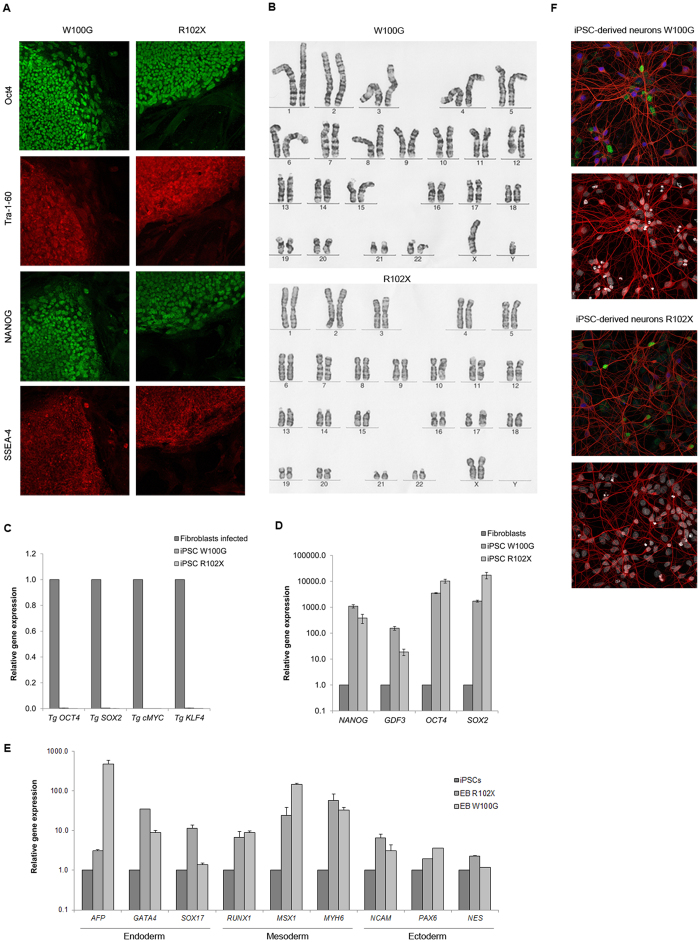
Characterization of iPSCs and cortical neurons derived from controls and M-D patients. (**A**) Immunofluorescence detection of pluripotency markers SSEA-4, NANOG, Tra-1-60, and OCT4 in iPSC colonies of both M-D patients. (**B**) Karyotype analysis of the iPSC clones from both patients. (**C**) Residual expression levels of the transgenes *OCT4, SOX2, cMYC*, and *KLF4* (relative to *ACTB*) used for retroviral reprogramming. Values were normalized for respective expression levels in infected fibroblasts (isolated 7 days post infection). The error bars indicate SD. (**D**) Relative gene expression of the pluripotency markers *NANOG, GDF3, OCT4*, and *SOX2* in fibroblasts and iPSCs. *ACTB* served as housekeeping gene. Expression levels of fibroblasts were set to 1. (**E**) Relative gene expression of *AFP, GATA4* and *SOX17* (endoderm), *RUNX1, MSX1* and *MYH6* (mesoderm) as well as *NCAM, PAX6* and *NES* (ectoderm), representing all three germ layers. *ACTB* served as housekeeping gene and spontaneously differentiated embryoid bodies of iPSCs were compared to the respective iPSC line. (**F**) Immunofluorescence analysis of the neuronal marker TUJ1 (red), and the cortical markers Tbr1 (blue) and Brn2 (green) (upper image) and DAPI (white) (lower image) in iPSC-derived patient neurons.

We generated iPSC colonies from fibroblasts of a patient with W100G *SGCE* and from a patient harbouring the most common *SGCE* mutation - R102X^10^. Immunofluorescence analysis indicated high levels of the endogenous pluripotency markers OCT4, Tra-1-60, NANOG, and SSEA-4 in the patient iPSC lines (Fig. 1A). The karyotype of both lines was normal (Fig. 1B) and quantitative RT-PCR analysis showed efficient silencing of the viral transgenes *OCT4, SOX2, cMYC,* and *KLF4* when compared to newly infected fibroblasts (Fig. 1C). In keeping with the immunofluorescence results, mRNA expression analysis indicated high levels of the pluripotency markers *NANOG, GDF3, OCT4*, and *SOX2* in both patient iPSC lines compared to non-transfected fibroblasts (Fig. 1D). Comparison of *AFP, GATA4* and *SOX17* (endoderm), *RUNX1, MSX1* and *MYH6* (mesoderm) as well as *NCAM, PAX6* and *NES* (ectoderm) expression by quantitative RT-PCR of plated embryoid bodies and the respective iPSCs confirmed the potential of the iPSCs to diverge into all three germ layers (Fig. 1E). From these results we concluded that the M-D patient fibroblasts were successfully reprogrammed into iPSCs.

In light of predominant *SGCE* expression in the cortex^8^, the *SGCE*-mutant and control iPSCs were differentiated into cortical neurons. Immunofluorescence analysis indicated that about a third of cells positive for the neuronal marker TUJ1 also express Tbr1 (which localizes to the cortical layers I, II/III, Vb, VI and the subplate) or Brn2 (which is present in the cortical layers I,II/III and Vb)^11^ (Fig. 1F).

### Differential methylation of the *SGCE* promoter in control and M-D patient iPSCs and iPSC-derived neurons

**Figure 2:**
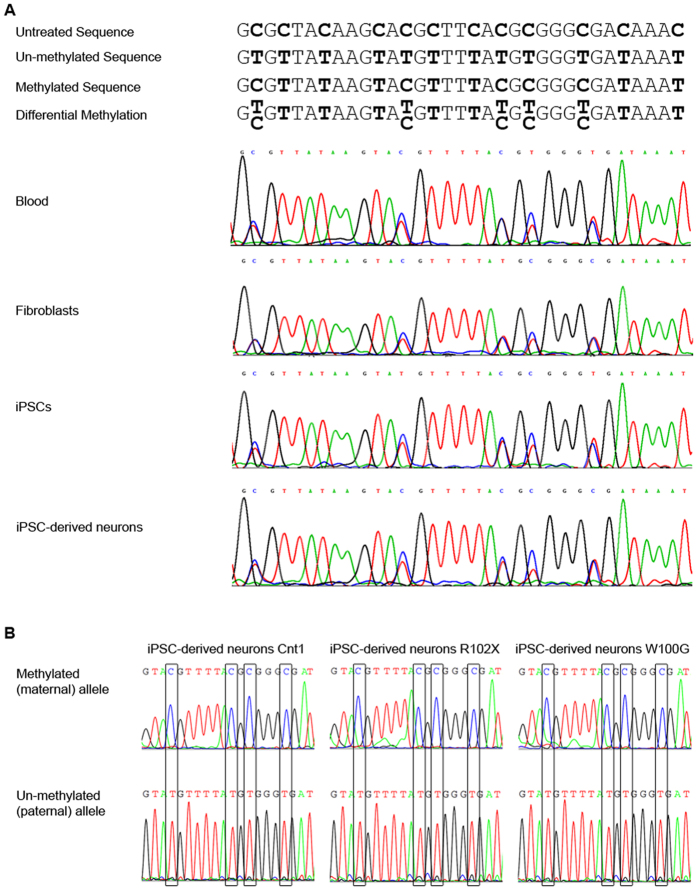
Detection of differentially methylated CpG dinucleotides in the promoter region of *SGCE* in iPSC-derived neurons. The methylation pattern of the *SGCE* promoter was investigated by DNA sequencing with methylation-specific primers after bisulfite treatment. Upon bisulfite treatment, un-methylated cytosines are converted to uracil and therefore appear as thymines in the resulting sequence. (**A**) Possible sequencing outcomes and their interpretation are illustrated in the upper panel. In the lower panel, sequencing results of DNA extracted from blood, fibroblasts, iPSCs and iPSC-derived neurons of a healthy individual are shown. Differential methylation was detected in all tissues. (**B**) Methylation-specific sequencing of the *SGCE* promoter region in iPSC-neurons of one control and both M-D patients revealed the presence of fully methylated DNA alongside fully unmethylated DNA in the samples representing the maternal and paternal allele, respectively. Continuous methylation (i.e. no alternation of non-methylated and methylated CpG islands) was detected.

Next, we investigated the impact of the reprogramming and differentiation procedure on the methylation status of the *SGCE* promoter. For this, DNA was extracted from blood, fibroblasts, iPSCs, and iPSC-derived neurons of a healthy individual. Sanger sequencing after bisulfite treatment (which mediates the conversion of all un-methylated cytosines into uracil residues that subsequently appear as thymines) revealed differentially methylated CpG dinucleotides in all investigated control DNA samples (Fig. 2A). The same experiment was performed with DNA from the M-D patient neuron cultures. This analysis confirmed differential methylation at all of the 25 CpG repeats tested (data not shown).

Moreover, a sequencing approach with methylation-specific primers applied to bisulfite-treated DNA extracted from control (Cnt1) and M-D patient (W100G and R102X) iPSC-derived neurons indicated two distinct statuses of the promoter region – i.e. (i) methylation of all CpG islands along the entire length of the amplified DNA fragment or (ii) no occurrence of DNA methylation at all. Areas of intermittent methylation were not detected with our approach (Fig. 2B).

### iPSC-derived neurons from M-D patients and controls express the brain-specific splice variant of *SGCE*

**Figure 3:**
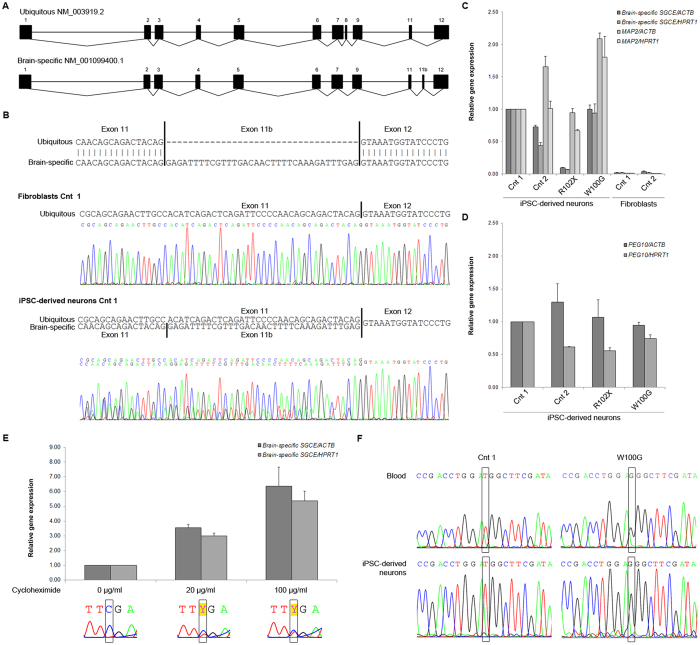
*SGCE* transcripts in fibroblasts and iPSC-derived neurons. (**A**) Sequence alignment of the ubiquitous isoform (NM_003919.2) and the brain-specific isoform (NM_001099400.1). The two isoforms differ with respect to the presence of exons 8 and 11b. (**B**) cDNA sequencing indicates expression of the ubiquitous mRNA *SGCE* isoform in fibroblasts and the presence of the ubiquitous as well as the brain-specific isoform (includes exon 11b) in iPSC-derived neurons. (**C**) Gene expression analysis in iPSC-derived neurons from M-D patients and controls. The levels of the brain-specific *SGCE* transcript and the neuronal marker *MAP2* were determined relative to the expression of the housekeeping genes *ACTB* and *HPRT1*. Fibroblasts from two healthy individuals were used as negative controls. Values were normalized to the neuronal control (Cnt) 1. The error bars indicate SE. (**D**) *PEG10* expression analysis in iPSC-derived neurons from M-D patients and controls. The levels of *PEG10* were determined relative to the expression of the housekeeping genes *ACTB* and *HPRT1*. The error bars indicate SE. (**E**) Expression of R102X brain-specific *SGCE* upon treatment with cycloheximide. Values were normalized to the nonsense mutant without treatment. The lower panel depicts the cDNA sequence upon different cycloheximide concentrations. (**F**) *SGCE* cDNA sequencing in blood and iPSC-derived neurons of the missense-mutant M-D patient (W100G). The absence of the maternal c.298T allele and selective expression of the paternal c.298G allele was in concordance with imprinting of the maternal wildtype allele in the patient neurons.

To further characterize the iPSC-derived neurons from M-D patients with *SGCE* mutations and controls, we analysed the two most abundant mRNA *SGCE* isoforms. The prevalent brain-specific isoform (NM_001099400.1) can be distinguished from the ubiquitous isoform (NM_003919.2) by the presence of the alternatively spliced exon 11b and the absence of exon 8 (Fig. 3A)^12^.

cDNA sequencing revealed that, while the ubiquitous *SGCE* isoform prevailed in control fibroblasts, a combination of ubiquitous and brain-specific *SGCE* was expressed in iPSC-derived neurons (Fig. 3B). Real-time PCR expression analysis with one primer situated in exon 11b supported this result. Markedly higher levels of brain-specific *SGCE* were detected in control and W100G-mutant iPSC-derived neurons compared to the levels in control fibroblasts. In neurons with the R102X *SGCE* nonsense mutation, however, a drastic reduction of the brain-specific *SGCE* isoform was observed. We obtained similar *SGCE* gene expression results irrespective of which housekeeping gene (*ACTB* or *HPRT1*) was used as reference (Fig. 3C). Furthermore, quantitative RT-PCR analysis of the marker gene *MAP2* confirmed the neuronal character of the resulting cell cultures (Fig. 3C).

### Reduced levels of brain-specific *SGCE* transcript in R102X-mutant patient neurons due to nonsense-mediated mRNA decay

First, to test whether the observed reduction in mRNA levels in the R102X neurons is specific to the brain-specific *SGCE* transcript, we also quantified the expression of *Paternally Expressed Gene 10 (PEG10*) in our neuronal samples. The maternally imprinted *PEG10* gene is located in a head-to-head position with *SGCE* and it shares some of its promoter region with *SGCE*^13^. Our quantitative RT-PCR analysis indicated no depletion of *PEG10* mRNA in neurons harbouring the R102X mutation in *SGCE* (Fig. 3D).

Next, we explored the possibility of nonsense-mediated mRNA decay (NMD) as cause of low *SGCE* transcript levels in the R102X neurons. Treating the cells with 0, 20 or 100 μg/ml cycloheximide - a potent NMD inhibitor^14^ - resulted in increasing mRNA concentrations of brain-specific *SGCE* (Fig. 3E, upper panel). Furthermore, cDNA sequencing at each of the different cycloheximide concentration steps implicated that the observed effect is predominantly due to elevated expression of the nonsense-mutant paternal allele after NMD inhibition (Fig. 3E, lower panel).

### Maternal imprinting of *SGCE* is maintained in iPSC-derived M-D patient neurons regardless of mutation type

Studying the mRNA in the iPSC-derived M-D patient neurons provided us with additional clues about the imprinting status of the *SGCE* promoter. Further exploiting our expression data, in the R102X neurons, drastically reduced *SGCE* levels were not only evidence of nonsense-mediated decay of mRNA encoded by the mutant paternal allele. Considering the previously observed ‘all-or-none’ methylation pattern of the *SGCE* promoter, reduced *SGCE* expression is also indicative of stable imprinting of the maternal wildtype allele throughout the reprogramming and differentiation procedure of the iPSCs.

This finding is supported by cDNA sequencing of *SGCE* in the missense-mutant neurons. In line with maternal imprinting, our analysis confirmed the presence of the paternal mutant c.298G allele (encoding a glycine at position 100 of ε-sarcoglycan) and complete absence of the maternal c.298T wildtype allele in the missense-mutant neurons (Fig. 3F).

### Wildtype ε-sarcoglycan localizes to the plasma membrane in iPSC-derived neurons

**Figure 4:**
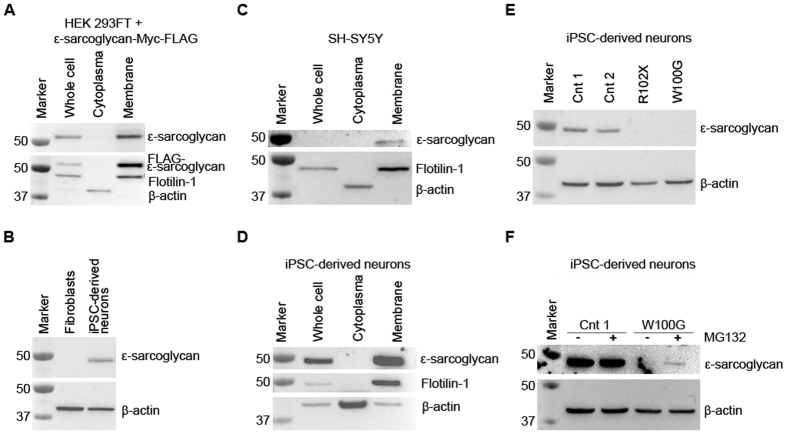
Cellular localization of ε-sarcoglycan in iPSC-derived cortical neurons. (**A**) The specificity of the esg2-1355 antibody was tested in HEK 293FT cells overexpressing brain-specific ε-sarcoglycan with a Myc-FLAG tag. In a fractionation experiment, ε-sarcoglycan-Myc-FLAG was selectively detected in the membrane fraction independently of the antibody used (anti-FLAG or esg2-1355). β-actin served as cytosolic marker, while Flotilin-1 was used to identify the membrane fraction. (**B**) Wildtype brain-specific ε-sarcoglycan was detectable in iPSC-derived control cortical neurons but not in fibroblasts using esg2-1355. β-actin protein levels were used as a loading control. (**C,D**) In SH-SY5Y cells (**C**) and iPSC-derived neurons (**D**), endogenous wildtype brain-specific ε-sarcoglycan was localized in the membrane fraction. (**E**) While endogenous brain-specific ε-sarcoglycan was identified in two control iPSC-derived neuron samples, no signal was observed in cells from the R102X nonsense mutant (due to nonsense-mediated mRNA decay). Similarly, endogenous W100G ε-sarcoglycan was undetectable in iPSC-derived patient neurons. (**F**) Treatment with the proteasome inhibitor MG132 partially rescued W100G ε-sarcoglycan in the cells.

In overexpression studies, ε-sarcoglycan has previously been shown to localize to the plasma membrane^9^. We were able to replicate this finding when transiently transfecting HEK 293FT cells with a vector expressing brain-specific ε-sarcoglycan-Myc-FLAG. In a fractionation experiment, efficient separation of the cytosol from the plasma membrane was achieved by differential centrifugation as indicated by the localization of the marker proteins β-actin and Flotilin-1 to the respective fractions (Fig. 4A). FLAG-tagged ε-sarcoglycan was selectively identified in the plasma membrane fraction using an anti-FLAG antibody as well as an antibody (esg2-1355) directed against the brain-associated isoform of ε-sarcoglycan (Fig. 4A) confirming the specificity of the latter antibody. Accordingly, the endogenous brain-specific isoform of the protein was detected in iPSC-derived control neurons but not in fibroblasts of a healthy individual (Fig. 4B). Fractionation experiments with SH-SY5Y cells and control iPSC-neurons also indicated that endogenous human ε-sarcoglycan localizes to the plasma membrane (Fig. 4C,D).

### The W100G missense mutation promotes proteasomal degradation of endogenous ε-sarcoglycan

Finally, we investigated the abundance of endogenous brain-specific ε-sarcoglycan in iPSC-derived neurons from the M-D patients with the R102X and W100G mutations, respectively. For both mutants, we did not detect any ε-sarcoglycan protein (Fig. 4E). For R102X, this is in line with NMD of mRNA transcribed from the mutated, paternal *SGCE* allele. In case of W100G ε-sarcoglycan, we hypothesized that the absence of the protein (despite high expression at the mRNA level) may be due to proteasomal degradation as previously observed in heterologous cells^9^,^15^. To prove this assumption, we treated missense mutant neurons with the proteasome inhibitor MG123 which resulted in a partial recovery of the abundance of W100G ε-sarcoglycan. By contrast, the treatment did not impact on the protein levels of wildtype ε-sarcoglycan in control iPSC-neurons (Fig. 4F). Importantly, the lack of ε-sarcoglycan protein in both patient samples is further evidence of complete imprinting of the maternal wildtype allele of *SGCE* in iPSC-derived neurons.

## Discussion

The objective of our study was to establish iPSC-derived cortical neurons from patients with mutations in the maternally imprinted gene *SGCE* as a suitable human cellular model system for M-D and – as proof-of-principle – for other neurological diseases where the epigenetic mechanism of imprinting plays an important role. Indeed, we have shown allele-specific methylation of the promoter region of *SGCE* in iPSC-derived neurons from healthy controls and mutation-positive patients for the first time. The *SGCE* imprinting patterns of the source fibroblast lines are maintained throughout the reprogramming and differentiation process. In control neurons, the brain-specific isoform of *SGCE* is expressed and the endogenous wildtype protein localizes to the plasma membrane. However, in the presence of the W100G missense mutation, ε-sarcoglycan is degraded intracellularly with involvement of the proteasome system. By contrast, the R102X mutation interferes with *SGCE* gene expression.

The advent of iPSC technology in 2006^16^ has revolutionized research especially in disorders affecting the brain. To date, well over a thousand studies have been published utilizing the original or adapted versions of the protocol by Takahashi and Yamanaka which allows conversion of somatic cells into a pluripotent state. The resulting iPSCs may be redirected into any desired cell type, including cortical neurons^17^,^18^.

Despite the unprecedented success of the method, concerns have recently been raised regarding non-physiological epigenetic alterations occurring during reprogramming which may interfere with the methylation status of imprinted genes^3^. Hypomethylation as the consequence of repeated iPSC passaging has been described for various loci^19^,^20^. Ultimately, loss of allele-specific gene expression may cause phenotypes to prevail which are not related to the disease under investigation^3^. An approach to circumvent or at least minimize the impact of epigenetic variations in iPSCs is to focus on ‘local’ phenotypes that can safely be traced back to a specific gene mutation. *SGCE*-associated M-D can serve as such an exemplary model, allowing the study of mechanisms of reduced penetrance which is a research field currently gaining considerable interest.

Extending our previous results from blood DNA^7^, we now showed that *SGCE* is maternally imprinted in iPSC-derived neurons from M-D patients with *SGCE* mutations. Differential methylation of the *SGCE* promoter region together with the absence of wildtype *SGCE* mRNA and/or ε-sarcoglycan protein provided evidence of epigenetic silencing of the maternal allele in missense and nonsense-mutant neurons. In light of a previous study demonstrating maternal imprinting of *SGCE* throughout the human brain^8^, we assume that iPSC-derived M-D patient cortical neurons mirror the physiological *SGCE* methylation status in the cortex.

Nevertheless, based on neuroimaging, the ‘all-or-none’ effect of *SGCE* imprinting has been questioned. When using positron emission tomography, metabolic changes were found in the thalamus, pons and cortex of M-D patients as well as non-manifesting individuals with mutations in the maternal *SGCE* allele^21^. To challenge these divergent hypotheses, it will be interesting to study *SGCE* promoter methylation in iPSC-derived neurons from clinically unaffected mutation-positive relatives of patients in the future.

*SGCE* mRNA transcripts are spliced in a tissue-specific manner. An isoform which includes an additional exon between exons 11 and 12, i.e. exon 11b, is predominantly expressed in the brain with highest levels in the motor cortex and somatosensory cortex^8^. Highlighting the fidelity of our M-D model system, we detected significantly increased cDNA concentrations of transcripts containing exon 11b in iPSC-derived neurons compared to fibroblasts. Further strengthening these results, an antibody directed against brain-specific ε-sarcoglycan identified the protein in iPSC-derived neurons but not in fibroblasts of controls.

The majority of M-D patients with a mutation in *SGCE* harbour deletions, insertions or nonsense changes that result in a premature stop codon^10^. As an example of such a scenario, we investigated iPSC-derived neurons from a patient carrying the R102X mutation. In these cells, mRNA levels of *SGCE* exon 11b were markedly reduced implicating nonsense-mediated decay of the mutant transcripts. Treatment of R102X neurons with the NMD inhibitor cycloheximide indeed confirmed that the mutation induces degradation of *SGCE* transcripts. Furthermore, expression analysis of *PEG10* – a gene which is located in a head-to-head position with *SGCE* and which is under the control of the same promoter^13^ – confirmed that the observed mRNA downregulation in the nonsense-mutant neurons is not a global phenomenon but instead limited to *SGCE* transcripts. In line with NMD, endogenous R102X ε-sarcoglycan protein was equally lacking in the patient neurons.

Overexpression of mouse wildtype or H36P, H36R, and L172R ε-sarcoglycan (corresponding to human H60P, H60R, and L196R ε-sarcoglycan, respectively) in neuronal human cell lines revealed that the normal protein localizes to the plasma membrane and the Golgi apparatus, whereas missense mutants are retained in the endoplasmic reticulum (ER). Assisted by the ubiquitin system, these newly synthesized misfolded ε-sarcoglycan forms undergo retrotranslocation from the ER to the proteasome^9^. Having access to fibroblasts from an M-D patient with a missense mutation, we tested the cellular abundance and localization of W100G ε-sarcoglycan in iPSC-derived cortical neurons and indeed observed phenotypes in line with the published data for mouse H36P, H36R, and L172R ε-sarcoglycan. At the endogenous level, W100G ε-sarcoglycan proved to be a target for proteasomal degradation in the cytosol.

Taken together, our characterization of iPSC-derived cortical neurons with mutations in *SGCE* revealed that these cells are a suitable model mirroring the endogenous environment in the M-D patient brain, especially, when focusing on concrete molecular aspects of the disease mechanism. We predict that future studies applying this model system will contribute to a better understanding of endogenous ε-sarcoglycan function and, more generally speaking, will further prompt the use of iPSC-derived cellular models to study epigenetic mechanisms impacting on health and disease.

## Methods

### Patients

All patients and control individuals gave informed consent and the Ethics Committee at the University of Lübeck approved the study. Further, all methods were performed in accordance with the experimental protocols approved by the Ethics Committee at the University of Lübeck. The diagnosis of M-D was established based on published criteria^10^. Detailed demographic and phenotypic information on the patients (L-5007 and L-6074) has been previously published^22^. Two healthy controls included in the study were aged 33 and 59 years at biopsy taking and carried no mutation in the *SGCE* gene.

### Culture of cell lines and iPSC-derived neurons

HEK 293FT cells, SH-SY5Y cells, and human dermal fibroblasts from controls and two M-D patients with mutations in *SGCE* (i.e. W100G and R102X) were grown at 37 °C under a 5% CO_2_ humidified atmosphere in DMEM supplemented with 10% FBS and 1% penicillin/streptomycin. Generation of iPSCs was carried out as previously published^1^. Cortical neuron differentiation was adapted from Shi *et al*.^18^. In brief, iPSCs were plated as single cells. At 95% confluency, differentiation was initiated in neural differentiation medium supplemented with Dorsomorphine (1 μM), SB 431542 (10 μM) and Y-27632 (10 μM). Until day twelve of differentiation, the medium composition was gradually shifted from neural differentiation medium to neural maintenance medium (NMM) supplemented with Dorsomorphine, SB 431542 and Y-27632. From day 13 to 17, the cells were cultured in NMM medium containing FGF (FGF2, basic fibroblast growth factor; 20 ng/ml) and BDNF (Brain-derived neurotrophic factor; 20 ng/ml). Neural rosettes were manually replated on day 18 in NMM with BDNF (20 ng/ml), GDNF (Glial cell-derived neurotrophic factor; 20 ng/ml), and AA (ascorbic acid; 0.2 mM). The medium was exchanged every second day until day 27 including an additional manual rosette replating on day 23. On day 28, the rosettes were dissociated using accutase and plated at desired densities in NMM (with BDNF, GDNF, and AA). Until day 43, the medium (NMM with BDNF, GDNF, and AA) was changed every three days. For final differentiation the cells were cultured in NMM.

Inhibition of NMD was achieved by cycloheximide treatment for 8 hours at 20 μg/ml and 100 μg/ml final concentration. Proteasome inhibition by MG132 treatment was carried out at 10 μM final concentration for 8 hours. For transient transfection of brain-specific *SGCE* in HEK 293FT cells, a pCMV6 Entry vector (OriGene Technologies, Inc. Rockville, USA) was used. Cells for immunofluorescence were fixed in 4% formaldehyde for 15 minutes, permeabilised with 0.1% Triton X-100, and blocked in 4% appropriate normal serum.

### Western blotting and immunofluorescence analyses

Western blotting and immunofluorescence analyses were performed as published^23^ with the following antibodies: anti-OCT4 (Abcam, Cambridge, UK), anti-Tra-1-60 (Merck Millipore, Darmstadt, Germany), anti-NANOG (Stemgent, Lexington, USA), anti-SSEA-4 (Merck Millipore, Darmstadt, Germany), anti-TUJ1 (Covance Inc., Princeton, USA), anti-Tbr1 (Abcam, Cambridge, UK), anti-Brn2 (Santa Cruz), anti-ε-sarcoglycan (esg2-1355, published antibody against the brain-specific isoform of the protein)^24^, anti-FLAG (Sigma Aldrich, St. Louis, USA), anti-Flotilin-1 (Cell Signaling Technology, Danvers, USA), and anti-β-actin (Sigma Aldrich, St. Louis, USA).

### RNA extraction, real-time PCR analysis, and sequencing

RNA was extracted using the RNAeasy protect kit (Qiagen, Venlo, Netherlands). Complementary DNA was synthesized with the Maxima First Strand cDNA Synthesis Kit (Thermo Fisher Scientific, Waltham, USA). Quantification was carried out on the LC480 (Roche) system with the Maxima SYBR Green/Fluorescein qPCR Master Mix (Thermo Fisher Scientific, Waltham, USA). Bisulfite treatment of DNA was performed with the Premium Bisulfite Kit (Diagenode, Liège, Belgium). DNA sequences were obtained by Sanger sequencing on an ABI 3130XL system (Applied Biosystems; Thermo Fisher Scientific, Waltham, USA). To test whether imprinting occurs on a single *SGCE* allele only, DNA was extracted from control and patient neurons and treated with bisulfite. Using this DNA, methylation-specific amplification of the promoter region of *SGCE* was achieved by primers binding selectively to regions containing multiple CpG islands. The resulting sequences represent pooled neuronal DNA enriched for either the presence or absence of methylated sites. All primer sequences can be found in the Supplementary Table S1.

### Subcellular fractionation

Cell pellets were dissolved in a homogenization buffer (sucrose 250 mM, Hepes 10 mM, pH 7.4) with anti-protease cocktail at 4 °C. Lysis was achieved by 30 strokes through a G22 needle and a 1 ml syringe. Through consecutive steps of centrifugation (10 min at 1,000 g and 10 min at 10,000 g), whole cells, debris and mitochondria were extracted from the solution. The membrane fraction and the cytosol were separated after 3 hours of centrifugation at 18,000 g^25^.

## Additional Information

**How to cite this article**: Grütz, K. *et al*. Faithful *SGCE* imprinting in iPSC-derived cortical neurons: an endogenous cellular model of myoclonus-dystonia. *Sci. Rep.* **7**, 41156; doi: 10.1038/srep41156 (2017).

**Publisher's note:** Springer Nature remains neutral with regard to jurisdictional claims in published maps and institutional affiliations.

## Supplementary Material

Supplementary Table S1
