# Supplementary material for: Faithful SGCE imprinting in iPSC-derived cortical neurons: an endogenous cellular model of myoclonus-dystonia
Source: Sci Rep. 2017 Feb 3;7:41156. doi: 10.1038/srep41156 (PMC5290732; doi:10.1038/srep41156)
Supplement: Supplementary Table S1 [file srep41156-s1.pdf]

Supplementary Information

Faithful *SGCE* imprinting in iPSC-derived cortical neurons: an endogenous cellular model of myoclonus-dystonia

Karen Grütz, MSc; Philip Seibler, PhD; Anne Weissbach, MD; Katja Lohmann, PhD; Francesca A. Carlisle, BSc; Derek J. Blake, DPhil; Ana Westenberger, PhD; Christine Klein*, MD; Anne Grünewald, PhD

Corresponding author:

Christine Klein, MD

Christine.klein@neuro.uni-luebeck.de

Institute of Neurogenetics, University of Lübeck, Lübeck, Germany

Supplementary Table S1 Primers for quantification of cDNA, PCR-amplification and sequencing of bisulfite-treated genomic DNA, and PCR-amplification and sequencing of *SGCE* cDNA

Gene	Template	Application	Forward 5‘-3‘	Reverse 5‘-3‘
ACTB	cDNA	qPCR	TGAAGTGTGACGTGGACATC	GGAGGAGCAATGATCTTGAT
HPRT1	cDNA	qPCR	ATCAGACTGAAGAGCTATTGTAATGACCA	TGGCTTATATCCAACACTTCGTG
OCT4 endo [1]	cDNA	qPCR	CCTCACTTCACTGCACTGTA	CAGGTTTTCTTTCCCTAGCT
OCT4 trans [1]	cDNA	qPCR	CCTCACTTCACTGCACTGTA	CCTTGAGGTACCAGAGATCT
SOX2 endo [1]	cDNA	qPCR	CCCAGCAGACTTCACATGT	CCTCCCATTTCCCTCGTTTT
SOX2 trans [1]	cDNA	qPCR	CCCAGCAGACTTCACATGT	CCTTGAGGTACCAGAGATCT
KLF4 endo [1]	cDNA	qPCR	GATGAACTGACCAGGCACTA	GTGGGTCATATCCACTGTCT
KLF4 trans [1]	cDNA	qPCR	GATGAACTGACCAGGCACTA	CCTTGAGGTACCAGAGATCT
cMYC endo [1]	cDNA	qPCR	TGCCTCAAATTGGACTTTGG	GATTGAAATTCTGTGTAACTGC
cMYC trans [1]	cDNA	qPCR	TGCCTCAAATTGGACTTTGG	CGCTCGAGGTTAACGAATT
NANOG [1]	cDNA	qPCR	TGAACCTCAGCTACAAACAG	TGGTGGTAGGAAGAGTAAAG
GDF3 [1]	cDNA	qPCR	AAATGTTTGTGTTGCGGTCA	TCTGGCACAGGTGTCTTCAG
GATA4 endoderm [1]	cDNA	qPCR	CTAGACCGTGGGTTTTGCAT	TGGGTTAAGTGCCCCTGTAG
AFP endoderm [1]	cDNA	qPCR	AGCTTGGTGGTGGATGAAAC	CCCTCTTCAGCAAAGCAGAC
SOX17 endoderm	cDNA	qPCR	CTCTGCCTCCTCCACGAA	CAGAATCCAGACCTGCACAA
RUNX1 mesoderm [1]	cDNA	qPCR	CCCTAGGGGATGTTCCAGAT	TGAAGCTTTTCCCTCTTCCA
MSX1 mesoderm	cDNA	qPCR	CGAGAGGACCCCGTGGATGCAGAG	GGCGGCCATCTTCAGCTTCTCCAG
MYH6 mesoderm	cDNA	qPCR	TCAGCTGGAGGCCAAAGTAAAGGA	TTCTTGAGCTCTGAGCACTCGTCT
NCAM ectoderm [1]	cDNA	qPCR	ATGGAAACTCTATTAAAGTGAACCTG	TAGACCTCATACTCAGCATTCCAGT
PAX6 ectoderm	cDNA	qPCR	GTCCATCTTTGCTTGGGAAA	TAGCCAGGTTGCGAAGAACT
NES ectoderm [1]	cDNA	qPCR	GCGTTGGAACAGAGGTTGGA	TGGGAGCAAAGATCCAAGAC
MAP2	cDNA	qPCR	CAGGTGGCGGACGTGTGAAAATTGAGAGTG	CACGCTGGATCTGCCTGGGGACTGTG
PEG10	cDNA	qPCR	TGTCGTCCGGAGCCACTC	AGGGTGACGGTTGGGGTG
Brain-specific SGCE	cDNA	qPCR	GGAGATTTTCGTTTGACAAC	CAACATGCATAACATATGCCAG
SGCE PromM [2]	gDNA	PCR, Seq	GTGTTATGTTTTATAAATAGATAAG	CAACTCATATACCTCTACAATTC
SGCE Prom Un-methylated	gDNA	PCR, Seq	TTTTATAAATAGATAAGTGTTTTAAATGT	CAACTCATATACCTCTACAATTC
SGCE Prom Methylated	gDNA	PCR, Seq	TTTATAAATAGATAAGCGTTTTAAATGC	ACTACGACTACTTTATTTATAACGCAAA
SGCE whole transcript [3]	cDNA	PCR, Seq	CTGATGCTGAACTGGCCAAG	CAACATGCATAACATATGCCAG
SGCE Ex 3 internal [2]	cDNA	Seq	GCGAGATTAGTAATGATCCC	

endo - endogenous, trans - transgene, qPCR - quantitative PCR, Seq – Sequencing

1 Park, I. H. *et al.* Reprogramming of human somatic cells to pluripotency with defined factors. *Nature* **451**, 141-146, doi:nature06534 [pii] 10.1038/nature06534 (2008).

2 Muller, B. *et al.* Evidence that paternal expression of the epsilon-sarcoglycan gene accounts for reduced penetrance in myoclonus-dystonia. *Am J Hum Genet* **71**, 1303-1311 (2002).

3 Xiao, J., Nance, M. A. & LeDoux, M. S. Incomplete nonsense-mediated decay facilitates detection of a multi-exonic deletion mutation in SGCE. *Clin Genet* **84**, 276-280, doi:10.1111/cge.12059 (2013).
